# Delayed Chylothorax during Treatment of Follicular Lymphoma with a Malignant Pleural Effusion

**DOI:** 10.1155/2020/2893942

**Published:** 2020-02-25

**Authors:** Chigozirim N. Ekeke, Ernest G. Chan, James D. Luketich, Rajeev Dhupar

**Affiliations:** ^1^Department of Cardiothoracic Surgery, University of Pittsburgh, Pittsburgh, PA, USA; ^2^Surgical Services Division, Veterans Affairs Pittsburgh Healthcare System, Pittsburgh, PA, USA

## Abstract

Chylothorax occurs following dysfunction or disruption of the lymphatic drainage along the thoracic duct. Malignant and traumatic causes account for the majority of these occurrences, with lymphoma accounting for 11-37% of chylothoraces. The clinical course of chylothorax may include dehydration, malnutrition, immunosuppression, electrolyte disturbances, infection, and ultimately death. Management of chylothorax is patient-specific and is based on etiology and surgeon experience. Initially, most chyle leaks are managed with nonoperative strategies, such as gut rest, hyperalimentation, and pleural drainage, and, at times, medium-chained fatty acid diet or octreotide, with hopes to decrease chyle production (Zabeck et al. (2011)). High-output chyle leaks following iatrogenic injury or trauma are commonly managed with thoracic duct ligation. Lymphangiography with or without thoracic duct embolization has become increasingly popular and efficacious with the possible benefit of less morbidity (Cope et al. (2002)). We report a case of a 61-year-old male with delayed chylothorax while having an indwelling pleural catheter for malignant pleural effusion during treatment of follicular lymphoma. Percutaneous thoracic duct embolization was attempted but was unsuccessful. Chemotherapy, fluid management, and nutritional support allowed this to resolve over the course of ninety days from diagnosis. We describe the patient's clinical course and highlight nonoperative management of delayed chylothorax in the setting of follicular lymphoma treatment.

## 1. Introduction

Chylothorax is the collection of lymph into the thoracic cavity, usually from the thoracic duct or its associated tributaries. It most commonly occurs following iatrogenic injury during surgery (e.g., esophagectomy, cardiac surgery, or central line placement) [1]. Other etiologies include trauma, malignancy, and idiopathy [2]. Chyle is comprised of lymphocytes, electrolytes, and triglycerides. The loss of large volumes of chyle can lead to metabolic disturbances, immunologic derangement, and sometimes death. Low-output chylothorax (<1 L/daily) is commonly managed with bowel rest, drainage of fluid for symptoms, and sometimes TPN. High-output (>1 L/daily) or refractory low-output chylothoraces are seen following esophagectomy, congenital cardiac surgery, or mediastinal node dissections, for which a different management strategy is generally undertaken, using surgical thoracic duct ligation or embolization.

Our case report describes a patient who presented with symptomatic malignant pleural effusion secondary to follicular lymphoma. An indwelling pleural catheter was in place to manage his symptoms, but during chemotherapy, he developed a chylothorax in a delayed fashion. He was managed with nonfat diet and octreotide, followed by lymphangiogram with thoracic duct embolization. Ultimately, it was the treatment of his lymphoma and patience that resulted in resolution. Delayed chylothorax is uncommonly described in the literature. This paper has been reported in line with the SCARE criteria [3].

## 2. Description

**Figure 1:**
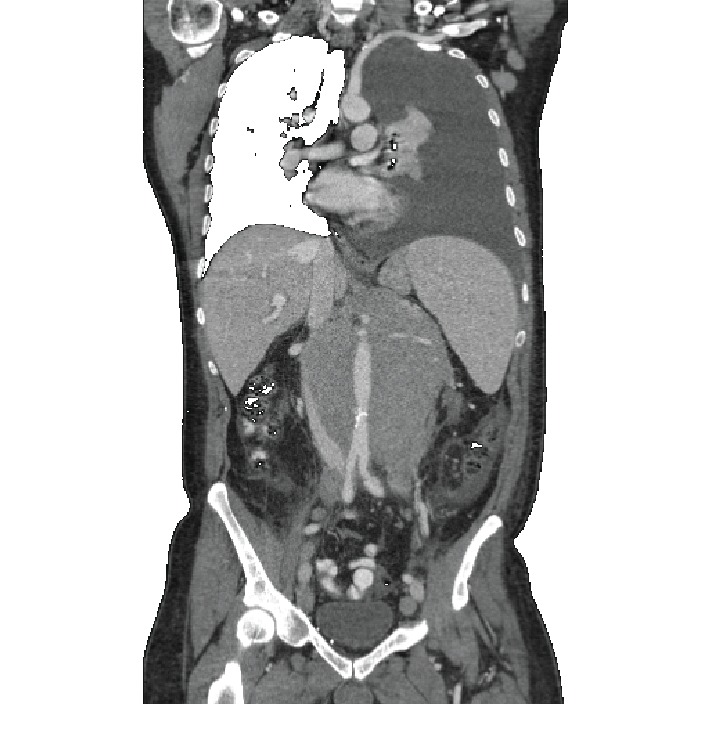
Coronal view of the chest with a large malignant pleural effusion.

A 61-year-old male presented with a 7-day history of dyspnea with exertion, productive cough, orthopnea, and declined daily activity. He had thrombocytopenia but no other laboratory abnormalities. Physical examination revealed palpable lymph nodes in the bilateral axilla and groins. His CT scan (Figure 1) showed a large left pleural effusion, mediastinal and retroperitoneal adenopathy, and large superficial right common femoral node. A 14-French chest tube was placed, resulting in improved symptoms after drainage of more than 1 liter of serous fluid, and cytologic evaluation revealed a mature B cell lymphoma. He underwent biopsy of a femoral lymph node which revealed follicular lymphoma.

He began chemotherapy, but his symptomatic effusion recurred. Therefore, an indwelling tunneled pleural catheter was placed, yielding serous fluid. He was treated with bendamustine and rituximab, with plans to restage his disease after three cycles. He was noted to have transient improvement in his symptoms, but he continued to drain 600-1500 cc/daily. Sixty days after his initial diagnosis, the pleural fluid character changed to a white-opaque consistency that was positive for chylomicrons and an elevated triglyceride value of 1600 mg/dL.

**Figure 2:**
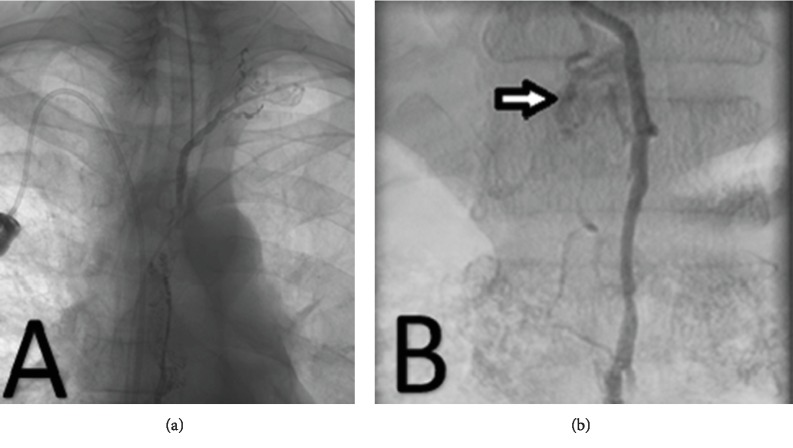
Thoracic (a) and abdominal (b) lymphangiogram delineating the thoracic duct using contrast dye; evidence of extravasation (arrow).

He was started on a nonfat diet and administered octreotide but continued to drain 1 liter of chyle daily as an outpatient. Thirty days following the diagnosis of chylothorax, interventional radiology was consulted for diagnostic and therapeutic intervention for the persistent chylothorax. He underwent lymphangiography with thoracic duct embolization (Figures 2(a) and 2(b)).

**Figure 3:**
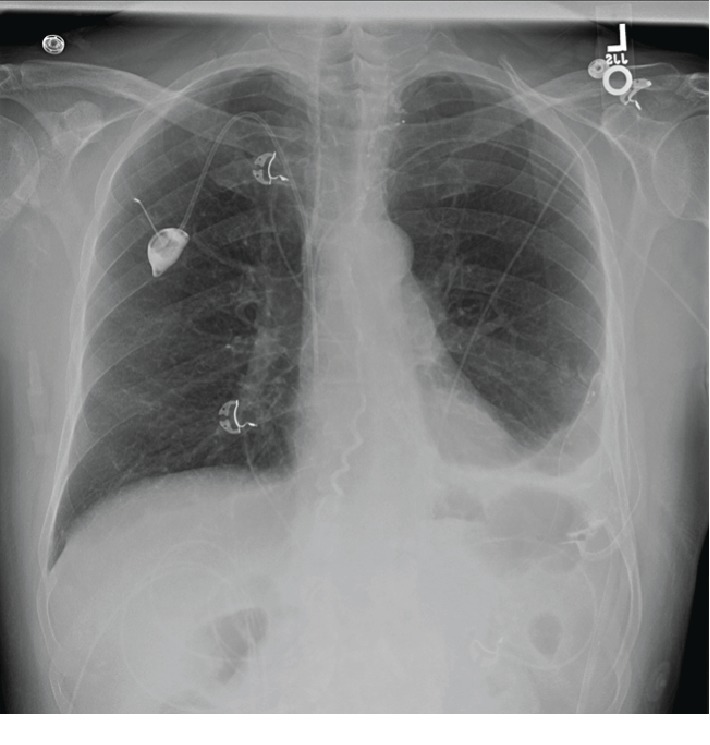
Chest X-ray after thoracic duct lymphangiogram using glue embolization, with resolving chylothorax in the left hemithorax.

Following the procedure, his drainage decreased to 600-1000 mL/day. Chest X-ray showed improved left pleural effusion, and he endorsed no shortness of breath 2 weeks after the procedure (Figure 3). Thirty days following his lymphangiography, he received intrapleural alteplase to drain any residual collection and completed his chemotherapy. His output continued to decrease, and his indwelling pleural catheter was removed 90 days after diagnosis of the chylothorax, 150 days after diagnosis of the malignant pleural effusion.

## 3. Discussion

In our report, we described managing a delayed chylothorax in the setting of malignant pleural effusion from lymphoma. After presenting with a large symptomatic pleural effusion and subsequently being diagnosed with B cell lymphoma, this patient underwent placement of an indwelling intrapleural catheter. His chemotherapeutic course was accompanied with a delayed onset of high-output chylothorax 60 days following diagnosis of the malignant pleural effusion. We attempted thoracic duct embolization given his long course of high output. Despite failed thoracic duct embolization, his lymphoma responded well to chemotherapy and the chyle leak stopped approximately 60 days following embolization.

His lymphoma ultimately responded to the chemotherapy, and his chylothorax resolved ninety days after diagnosis, and his catheter was removed. This is an unusual case in which our patient developed a malignant pleural effusion and was treated with chemotherapy and then developed a delayed high-output chylothorax.

In general, chylothorax is an uncommon cause of pleural effusion. Iatrogenic injury following thoracic intervention is the most common cause, while nontraumatic causes include malignancy, superior vena cava syndrome, sarcoidosis, tuberculosis, amyloidosis, congenital duct abnormalities, and diseases of the lymph vessels such as yellow nail syndrome and lymphangioleiomyomatosis. Lymphoma, chronic lymphocytic leukemia, and metastatic cancer are the more common etiologies of nontraumatic chylothorax, with resolution usually following chemotherapy or radiation [1, 4, 5]. Control of the underlying malignancy is still the mainstay of treatment and reported as the most effective. Surgical intervention in noniatrogenic cases is rarely performed, and the literature is limited regarding outcomes in the malignant cohort. Delayed chylothorax is rare but has been described most commonly with iatrogenic causes such as mediastinal lymph node dissection, heart-lung transplantation, or thoracic sympathectomy [6–8]. Doo et al. described a case of delayed chylothorax (26 years) following thoracic sympathectomy, which resolved successfully following thoracic duct ligation and pleurodesis [9].

The timing of thoracic duct intervention varies and remains controversial. Some advocate for immediate intervention with high outputs of chyle (>1 L/daily) on the first postoperative day, unchanged drainage over 48 hours, or clinical deterioration [10, 11]. Percutaneous thoracic duct embolization has been increasingly popular given its reported success rates of 70% at high-volume centers [12].

Thus far, there are no existing prospective trials delineating which intervention is best based on etiology of chylothorax. We advocate for dietary changes, symptomatic management of the effusion, and treatment of the underlying etiology for initial management, but percutaneous embolization and close monitoring for clinical decline in noniatrogenic causes of chylothorax. Specifically, lymphangiogram with embolization is a strategy to delineate the anatomy and severity of leak and potentially improve or eliminate the leak. A prospective, randomized controlled trial would need to be performed to accurately approve or disprove this approach; however, this is not feasible.
